# Intracardiac Echocardiography during Catheter-Based Ablation of Atrial Fibrillation

**DOI:** 10.1155/2012/921746

**Published:** 2012-05-29

**Authors:** Jürgen Biermann, Christoph Bode, Stefan Asbach

**Affiliations:** Department of Cardiology and Angiology, University Hospital of Freiburg, Hugstetter Stra*β*e 55, 79106 Freiburg, Germany

## Abstract

Accurate delineation of the variable left atrial anatomy is of utmost importance during anatomically based ablation procedures for atrial fibrillation targeting the pulmonary veins and possibly other structures of the atria. Intracardiac echocardiography allows real-time visualisation of the left atrium and adjacent structures and thus facilitates precise guidance of catheter-based ablation of atrial fibrillation. In patients with abnormal anatomy of the atria and/or the interatrial septum, intracardiac ultrasound might be especially valuable to guide transseptal access. Software algorithms like CARTOSound (Biosense Webster, Diamond Bar, USA) offer the opportunity to reconstruct multiple two-dimensional ultrasound fans generated by intracardiac echocardiography to a three-dimensional object which can be merged to a computed tomography or magnetic resonance imaging reconstruction of the left atrium. Intracardiac ultrasound reduces dwell time of catheters in the left atrium, fluoroscopy, and procedural time and is invaluable concerning early identification of potential adverse events. The application of intracardiac echocardiography has the great capability to improve success rates of catheter-based ablation procedures.

## 1. Introduction

Atrial fibrillation (AF) is the most prevalent sustained cardiac arrhythmia. Catheter-based ablation of AF is usually recommended for patients with symptomatic paroxysmal AF that is resistant to antiarrhythmic drug therapy. This approach is supported by results of randomized and prospective trials comparing antiarrhythmic drug treatment with catheter-based ablation, showing significantly better rhythm control after ablation. In addition, meta-analyses of studies performed mostly in patients with paroxysmal AF, comparing antiarrhythmic drugs and catheter-based ablation, have also clearly supported these findings [1–5].

As catheter-based ablation with electrical isolation of the pulmonary veins (PVs) has become an established therapeutic option for patients with symptomatic AF, accurate recognition of the complex and variable anatomy of the left atrium (LA) is indispensable. Circumferential PV isolation is generally guided by three-dimensional (3D) electroanatomical mapping [6–8], fluoroscopy [9], and/or intracardiac echocardiography (ICE) [10, 11]. However, only ICE offers the unique ability to image the LA in real time during the course of the procedure and to identify all structures which are important for the ablation.

Endpoints for a circumferential PV isolation procedure are either amplitude reduction within the ablated area [6, 8], elimination of the PV potentials recorded from the mapping catheter within the ipsilateral PV [7, 9, 10, 12–15], and/or exit block from the PV [16]. To achieve these endpoints in a safe and effective manner, ICE allows precise guidance of catheter-based ablations of arrhythmic foci in the PVs [17] and directly monitors, for example, tissue changes or development of microbubbles under ablation.

Intracardiac ultrasound is a complementary tool to fluoroscopy to guide safe transseptal access. It can be safely used throughout invasive procedures to early detect complications and possibly avoid adverse events. Furthermore, it has helped to improve anticoagulation regimes [18]. The use of ICE has improved success rate [19]. It may supplant fluoroscopy as the gold standard for precise imaging of endocardial structures during ablation of AF [20] and even catheter ablation of AF without fluoroscopy only using ICE and electroanatomic mapping has been successfully evaluated [21].

## 2. Technologies of Intracardiac Echocardiography

For visualisation of cardiac anatomy in general two different ICE technologies are available [22, 23]. The first one uses a phased-array ultrasound tipped catheter that consists of a 64-element transducer (AcuNav Ultrasound Catheter, Biosense Webster, manufactured by Siemens Medical Solutions, Diamond Bar, USA). The high-resolution, multiple-frequency transducer (5–10 MHz) is incorporated into a 10 F steerable catheter (four directions) and provides 90° sector images with depth control. This catheter allows for the whole spectrum of Doppler imaging capabilities. The second one is a mechanical ultrasound transducer-tipped catheter (ClearView, Cardiovascular Imaging Systems USA, Inc.) connected to an ultrasound machine (Boston Scientific Corporation USA). For intracardiac use, this catheter uses a 9 MHz single element transducer mounted at the tip of an 8 F catheter. A piezoelectric crystal rotates at 1800 revolutions per minute providing cross-sectional images in a 360° radial plane. Pulling the catheter within the heart, three-dimensional reconstruction of the anatomy can be obtained [24].

Sector imaging, flexibility in changing the frequency, and full Doppler capabilities make the use of the system with the phased-array ultrasound-tipped catheter preferable, especially in catheter-based ablation procedures. Therefore, the present paper will focus on the clinical application of this system (AcuNav Ultrasound Catheter, Biosense Webster, Diamond Bar, USA). Vascular access is obtained in most patients via the femoral vein by inserting an 11 F sheath for a 10 F ICE catheter or a 9 F sheath for an 8 F ICE catheter, which is subsequently advanced into the right atrium. The AcuNav ICE catheter provides high-resolution 2D images with a penetration ranging from 2 mm to 12 cm. This allows imaging of the LA with the ICE placed in the right atrium.

The use of ICE during interventional cardiac procedures has several advantages compared to transthoracic or transoesophageal echocardiography. In comparison to transoesophageal echocardiography, patient discomfort is less, general anaesthesia is not needed, and there is no risk of aspiration. Transthoracic echocardiography usually does not offer an adequate imaging quality to visualise detailed left atrial anatomy and also would require cumbersome positioning during the ablation procedure by an additional operator.

## 3. Visualisation of Cardiac Anatomy

### 3.1. Intracardiac Panorama

**Figure 1:**
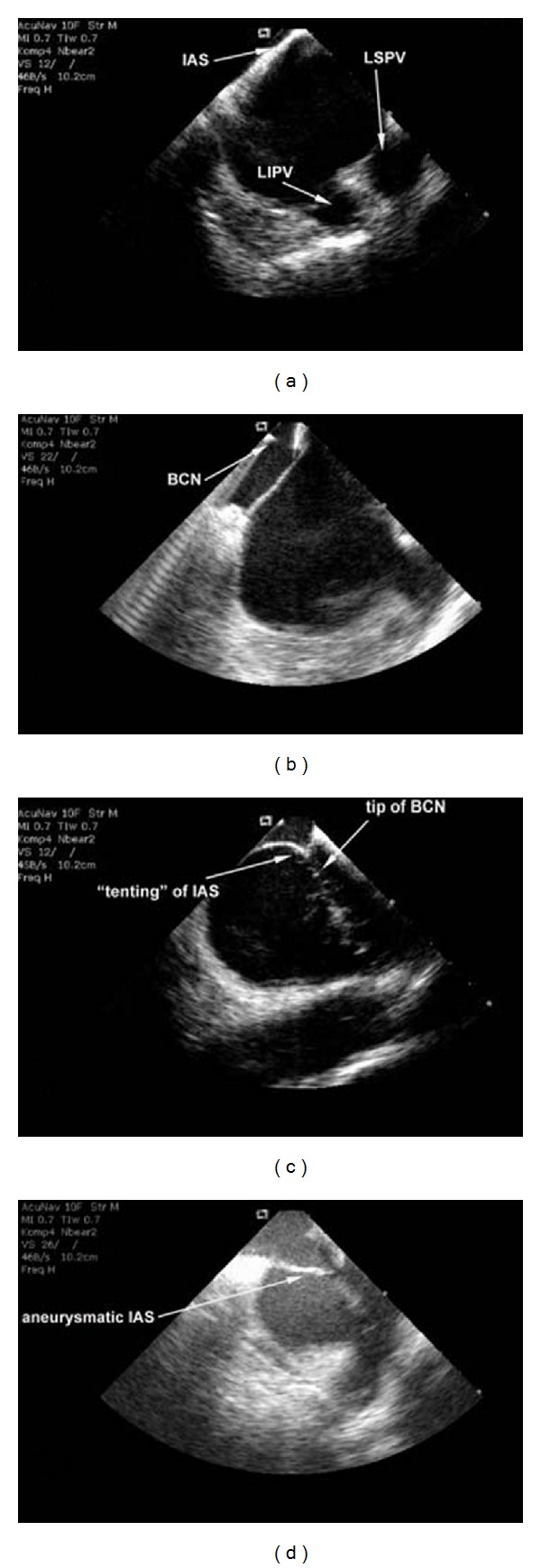
Visualisation of the LA and its adjacent structures with 2D ICE. (a) Interatrial septum (IAS), left superior pulmonary vein (LSPV), and left inferior pulmonary vein (LIPV). (b) The Brockenbrough-curved needle (BCN) is advanced from the right atrium to the IAS for transseptal puncture guided by ICE. (c) After successful transseptal puncture *tenting* of the IAS and the tip of the BCN in the LA are seen. (d) Example of abnormal anatomy of the IAS. An aneurysmatic IAS can hamper transseptal puncture. Progress of transseptal puncture can easily be visualised by 2D ICE.

The intracardiac position in the right atrium of the ICE catheter allows detailed visualisation of the LA due to close proximity to cardiac structures (Figure 1). This is of great importance, because accurate delineation of the variable anatomy of the LA and its adjacent structures is essential during an anatomically based approach of electrically isolating the PVs. Right atrium, right ventricle, and tricuspid valve are visualised in the so-called home view. By rotating the ICE catheter in a clockwise fashion the aortic valve, left ventricle and right ventricular outflow tract are in the field of view before the mitral valve and LA with the left atrial appendage appear. With more clockwise rotation the left superior pulmonary vein (LSPV) and left inferior pulmonary vein (LIPV) can be evaluated (Figure 1(a)). In a further posterior direction the oesophagus and right PVs are visualised. Intracardiac ultrasound also provides anatomic imaging of areas of special interest, for example, the Marshall ligament region or the lipomatous hypertrophy of the atrial septum. These hypertrophic structures can then be targeted for more aggressive energy delivery to create effective transmural PV isolation [25].

### 3.2. Crucial Anatomic Variations

The diameter of the LA is higher in patients with AF than that in patients without AF. Only in 68.6% of patients suffering from AF the anatomical pattern is typical with two right and two left PVs [26]. In comparison to the left-sided PVs, which frequently share a common ostium [27, 28], on the right side a right middle PV between the right superior pulmonary vein (RSPV) and right inferior pulmonary vein (RIPV) is common, accounting for 55% to 93% of accessory right-sided veins [29]. These anatomic variations are important in planning catheter ablation of AF. Localisation of the PV-LA boundary, the LA appendage, and the ridge between PV and LA appendage can be more accurate with assistance of ICE images acquired parallel to mapping and ablation.

Real-time visualisation of the LA and its adjacent structures is feasible and advantageous for online guiding of mapping and ablation catheters, not least because patients with AF often have atypical anatomical characteristics of the LA.

## 4. Guidance of Transseptal Access

### 4.1. Interatrial Septum

In patients with abnormal anatomy of the interatrial septum (IAS) especially (e.g., thick, double membrane or aneurysmatic floppy septum; patent foramen ovale or atrial septal defect; lipomatous hypertrophy of the septum; previous cardiac surgery with distorted anatomy or thickened septum; after device closure of an atrial septal defect) ICE might be a valuable option to guide safe transseptal access (Figures 1(b)–1(d)). The ICE-guided transseptal puncture can avoid potential life threatening complications including aortic puncture, pericardial tamponade, systemic arterial embolism, and perforation of the inferior vena cava [30].

### 4.2. Preparing Transseptal Puncture

Accurate visualisation of the fossa ovalis reduces the risk of complications. The goal of transseptal access is to cross the septum in the posterior region of the fossa ovalis. The more anterior portions of the septum are depicted by views that display the aorta. An anterior puncture is not only less safe but also directs the catheters towards the mitral annulus, left ventricle, and the left atrial appendage. This anterior approach can make manipulations of catheters for an AF ablation procedure difficult because of the posteriorly located ostia of the PVs. Preparing transseptal puncture a Brockenbrough-curved needle is inserted via a transseptal sheath and dilator system and advanced in the posterior region of the fossa ovalis (Figure 1(b)).

### 4.3. Traverse of Needle and Sheath

The first sign of a stable contact of the transseptal dilator at the IAS is *tenting* of the septum at this site. After successful puncture (Figure 1(c)), a coronary guide wire may be advanced into a left PV to stabilise the traverse of transseptal needle and sheath. After withdrawal of the Brockenbrough-curved needle a mapping or ablation catheter can be positioned in the LA via the transseptal sheath. Coronary guide wires and catheters can easily be visualised when entering the LA, which again helps to reduce fluoroscopy time. In many ablation procedures double transseptal access is needed and is eased by ICE.

After completing the ablation procedure, catheters and sheaths are retracted leaving residual atrial septal defects. Because of their small size such residual atrial septal defects after using 8F sheaths appear to be clinically insignificant and typically resolve completely in the course of a few months. However, the size of residual septal defects may significantly increase with repeated reinsertion of catheters/sheaths passing through the defect during the ablation procedure [31].

## 5. Three-Dimensional Reconstruction of Left Atrial Anatomy

Accurate recognition of the complex and variable anatomy of the LA and the adjacent structures can be achieved by CT and/or MRI prior and electroanatomical mapping, fluoroscopy, rotational angiography, and ICE during ablation procedures.

**Figure 2:**
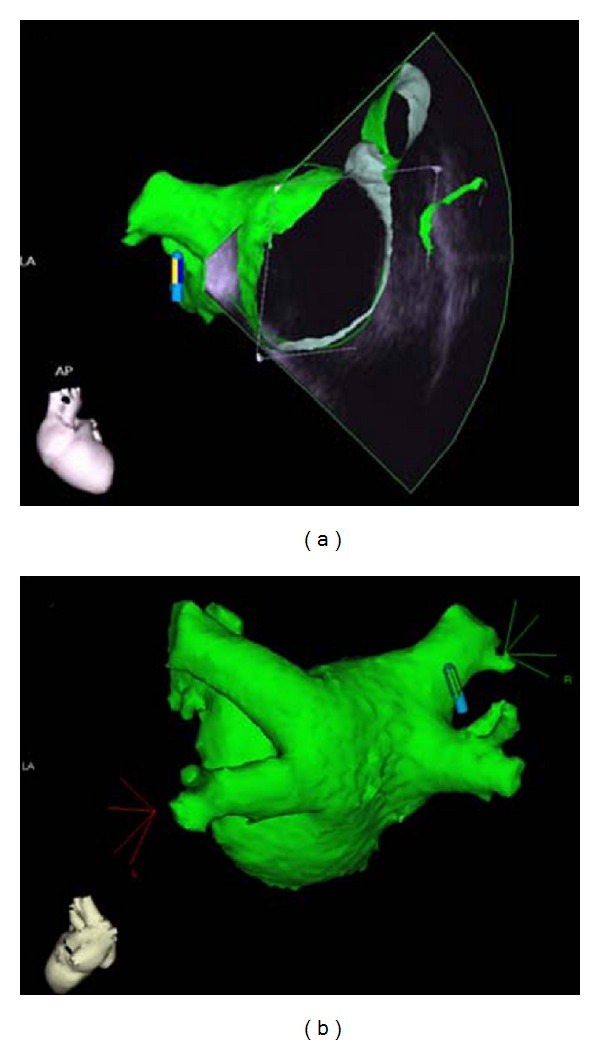
(a) ICE fan with overlying CT reconstruction of the LA in the anterior-posterior projection and (b) a completed reconstruction of the LA in posterior-anterior projection both using CARTOSound and the integrated software algorithm CARTOMerge.

The software algorithm CARTOSound (Biosense Webster, Diamond Bar, USA) offers the opportunity to reconstruct multiple 2D ultrasound fans generated by ICE to a 3D object (Figure 2) and has developed into a valuable tool for physicians guiding therapeutic cardiac interventions for almost all kinds of arrhythmias [32]. Creating a 3D shell of the LA from multiple 2D ICE images nowadays takes around 20 minutes [33, 34].

Ablation of AF through PV isolation is a challenging procedure. Success and associated complication rates are largely dependent on the operator and equipment. Due to the complex and varying anatomy of the LA, the LA-PV junction, and the PVs, general anatomical landmarks and fluoroscopic imaging offer vague guidance for left atrial ablation procedures. Image integration, usually with data acquired by CT or MRI of the LA, has thus been employed to accurately guide the safe electrical isolation of the PV ostia [32]. However, MRI has shortcomings associated with availability and costs, while cardiac CT causes additional radiation exposure for the patient and also requires contrast medium administration. The latter may be a problem in patients with chronic kidney insufficiency or hypersensitivity to contrast agents. In addition, both modalities come with the disadvantage of time intervals between imaging and the ablative procedure itself, during which changes of anatomical conditions and shape of the LA may occur, potentially due to changes in intravascular fluid volume, which result in an alteration of left atrial volumes and shape. The procedure of CT or MRI data registration itself might be subject to inaccuracies [35], which may be more marked in patients with dilated LA [36].

Integration of ICE imaging into a 3D reconstruction of the LA and relevant adjacent structures, along with electroanatomic data obtained with the mapping or ablation catheter, is a key upgrade to the utility of ICE for an AF ablation. Endocardial contours of the LA and the PVs can be obtained before transseptal puncture, by advancing the SoundStar 3D Ultrasound Catheter (Biosense Webster, Diamond Bar, USA) into the right atrium, the right ventricular outflow tract, and the coronary sinus, if necessary. Real-time imaging ensures correct assessment of the positions of relevant structures, including the oesophagus, which can be accurately outlined and integrated into the model of the LA. This may help to avoid inadvertent heat trauma during ablation. Since the first reports of its use in humans [37], the CARTOSound system has been employed for guiding ablation procedures, including, but not limited to AF, ablation procedures [38–40].

**Figure 3:**
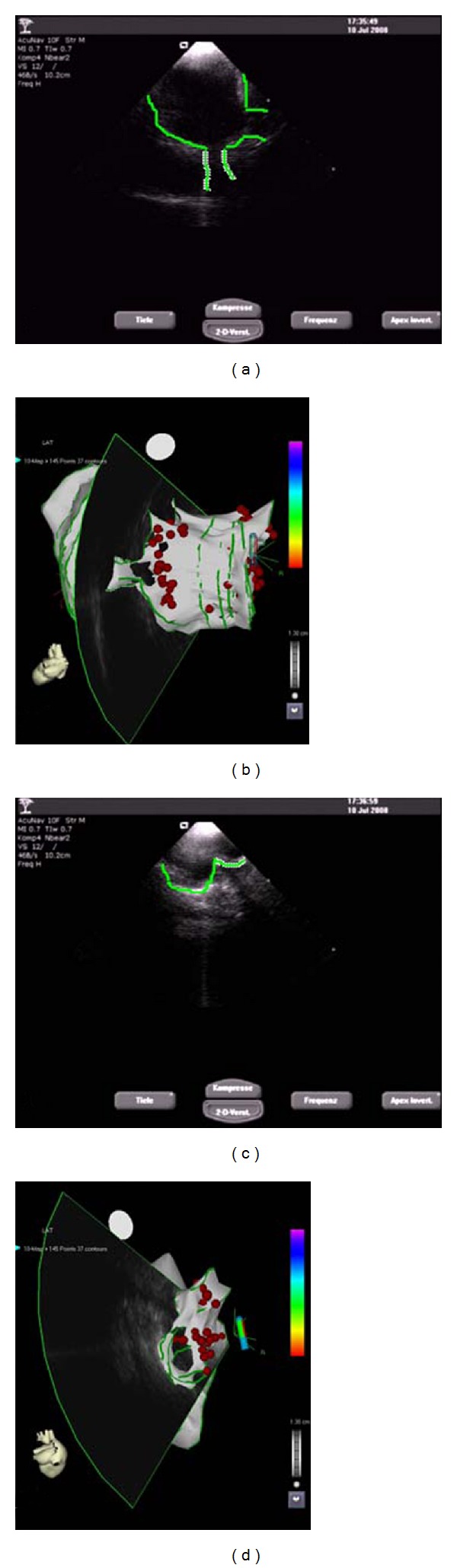
Reconstruction of left pulmonary veins (a and b) and right pulmonary veins (c and d) using ICE in combination with the integrated software algorithm CARTOMerge. A dedicated ICE probe provides real-time anatomic images that are integrated with preprocedural acquired CT images. On ECG-gated ICE images, the anatomy of the PVs can be annotated (a and c), creating a 3D shell, and subsequently is integrated with the dedicated algorithm (b and d).

**Figure 4:**
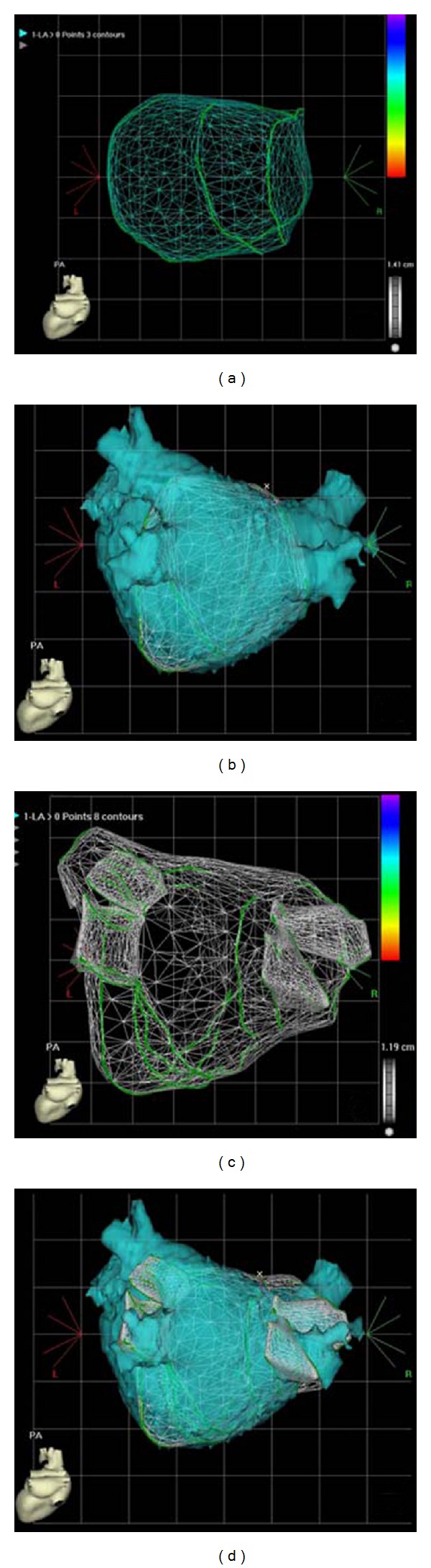
Three-dimensional ultrasound reconstruction of left atrial anatomy. (a) Minimal number of three ICE fans. (b) CARTOSound merge of three ICE fans with CT reconstruction of the LA. (c) Same LA with additional ICE fans of the body of the LA and all PVs. (d) CT reconstruction of the LA overlying all ICE fans (no further adjustment of LA orientation has been made).

The endocardial surface of the LA and its adjacent structures are traced in multiple ICE images creating several ICE fans of left atrial body, left atrial appendage, LSPV, LIPV, RSPV, and RIPV (Figure 3). The resulting 3D image of the LA can then be merged to a 3D CT or MRI reconstruction of the LA (Figure 4) using an integrated software algorithm (CARTOMerge, Biosense Webster, Diamond Bar, USA). Recording of only three ultrasound fans seems to be sufficient to exactly register the surface to a preprocedurally recorded CT or MRI [41]. The ablation procedure is subsequently guided by visualisation of the ablation catheter within the merged image, by intermittent fluoroscopy and continuous monitoring with ICE. Since ICE mapping can be performed from the right atrium, this approach involves shorter prothrombotic dwell time of catheters in the LA. Navigation of catheters is easier with potentially less fluoroscopy time [19, 41]. Intracardiac ultrasound integration into 3D electroanatomic reconstruction of the LA provides reliable guidance for PV isolation.

CARTOSound-guided PV isolation was studied with preprocedural MRI only or with the combination of MRI and/or ICE [42]. Total procedural time was similar between these three groups, but MRI integration required more fluoroscopy time and a longer dwell spent in the LA. Addition of MRI to ICE integration showed a tendency for a higher fluoroscopy time in comparison to ICE integration alone. Importantly, there were no significant differences in AF recurrences among these groups. Intracardiac ultrasound image integration significantly reduces fluoroscopy time and dwell in the LA, a parameter linked to the procedure-associated risk of cerebrovascular complications, in comparison to MRI integration alone.

Intracardiac ultrasound with 3D reconstruction appears to be a safe and effective alternative to MRI and CT data registration, although randomized comparisons are lacking. It is conceivable that this modality will see more widespread use in the near future, as the number of AF ablation treatments increases.

## 6. Visualisation and Navigation of Catheters

### 6.1. Real-Time Visualisation

For visualisation and navigation of mapping and ablation catheters, ICE is widely used in interventional cardiac electrophysiology. Advances in catheter-based ultrasound transducers and imaging technology provide meticulous images and enable direct real-time visualisation of anatomical structures when performing catheter-based ablation procedures. With an ICE catheter, the same operator is enabled to perform both intracardiac imaging and manipulation of the mapping/ablation catheters.

### 6.2. Monitoring Catheter-Tissue Contact

Whereas ablation procedures can be guided by analysis of intracardiac electrocardiograms and fluoroscopy only, the catheter-endocardial tissue contact is not reliably visualised. It has been demonstrated that ICE can ensure adequate catheter-tissue contact in order to achieve transmural lesions [43] and can provide imaging of lesion morphologic changes including swelling, dimpling, crater formation, accelerated bubbles before popping-crater like lesion development, and increased echogenicity during or immediately after lesion deployment. Based on real-time ICE monitoring of lesion development, titration of energy power and/or duration can control lesion formation and prevent tissue overheating or structural perforation [31].

### 6.3. Ablation Strategy and Outcome

Intracardiac ultrasound can provide detailed information on the individual PV anatomy, such as the number of PVs, the presence of common ostia, and additional PVs, which may influence the ablation strategy in anatomical-based isolation procedures [44]. The ICE technique is safe with a negligible rate of complications and good patient tolerance. It allows improvement in success rate and decreases complications when compared with the fluoroscopy-only approach. As Marrouche et al. demonstrated, outcome in terms of AF-free survival after ostial PV isolation is improved by using ICE for displaying the catheters [10]; further improvement could be shown when radiofrequency energy titration is based on visualisation of microbubbles by ICE.

## 7. Identification of Potential Adverse Events

### 7.1. Thrombus Formation

Intracardiac ultrasound imaging is a valuable tool for early detection of complications during AF ablation procedures and consequently allows for earlier intervention [19]. Because of the risk of systemic embolism, evaluation of the presence of intracardiac thrombus by transoesophageal echocardiography before left-sided procedures is mandatory. However, in some patients ICE was able to detect thrombus formations stuck at LA structures despite inconspicuous initial transoesophageal echocardiography [45]. These cases illustrate the value of real-time imaging by ICE during invasive procedures.

**Figure 5:**
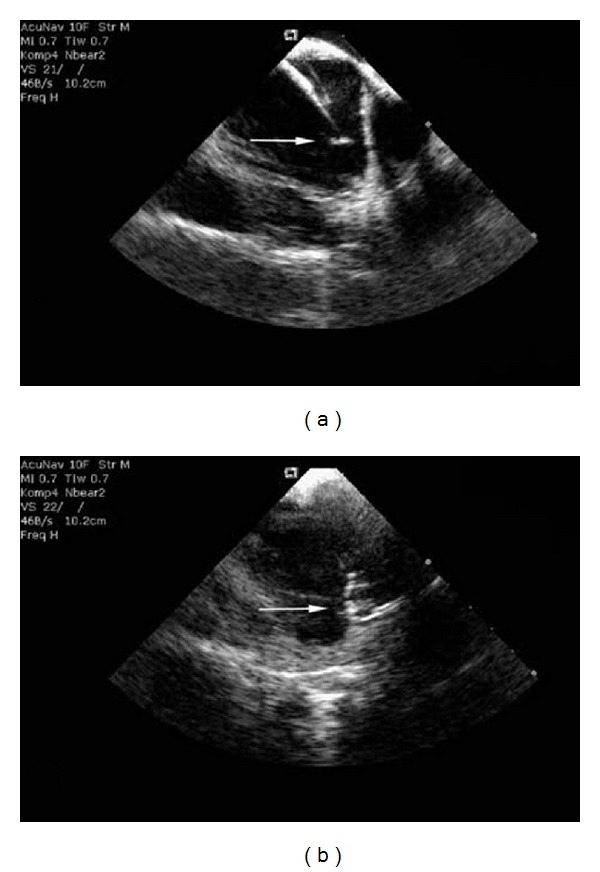
(a) A mobile thrombus (white arrow) is attached to the tip of the transseptal sheath in the LA before the advancement of a catheter and before heparin administration. (b) A circular mapping catheter can clearly be visualised in the antrum of the LSPV. Attached to the inferior part of the ring of electrodes a mobile thrombus (white arrow) can be detected (reproduction kindly permitted by the publisher [18]).

Usually thrombus formations form on the transseptal sheaths or the mapping/ablation catheters [18, 46] and are often found when activated clotting time is less than 300 seconds (Figure 5). While such findings may not affect specific acute intervention, they often have led to a change in anticoagulation protocols during the procedures. Administration of heparin before transseptal access is safe and minimises the risk of formation of clots on the sheaths or catheters [18, 19]. After successful transseptal puncture, the sheaths are flushed continuously with heparinized saline and the activated coagulation time is maintained between 300 and 400 seconds throughout the procedure [18]. In 90% of patients with real-time ICE-detected left atrial thrombus, successful withdrawal of the thrombus-attached catheters/sheaths from the LA into the right atrium has been reported to prevent any serious systemic embolic consequences [46].

### 7.2. Pericardial Effusion

Pericardial effusion can easily be detected and emergency intervention with pericardiocentesis guided by ICE can be performed. Pericardial effusion may occur immediately following catheter perforation of cardiac structures (e.g., interatrial septum, left atrial appendage, coronary sinus) or in the course of the ablation. Real-time ICE monitoring provides diagnosis of small pericardial effusions prior to hemodynamic changes and has been reported to allow earlier detection of a pericardial effusion than conventional echocardiography [47].

### 7.3. Pulmonary Vein Stenosis

Visualisation of microbubbles by ICE is an important characteristic according to which power settings of the radiofrequency energy delivery can be adjusted to ensure maximal energy delivery at the lowest risk of PV stenosis [10]. In animal studies it was observed that microbubbles appeared one to two seconds before an increase in impedance and temperature occurred [48]. Intracardiac Doppler colour flow measurements have been effectively used for monitoring PV ostial narrowing during focal AF ablation [49]. The fact that ablation targets, for example, the venoatrial junctions and the PVs or their ostia, are not easily visualised using fluoroscopy underlines the importance of ICE by preventing PV stenosis.

### 7.4. Left Atrial-Oesophageal Fistula

Left atrial-oesophageal fistula is a rare but feared possible major adverse event. Intracardiac ultrasound real-time imaging of the posterior LA wall contiguous to the oesophageal anterior wall has proven valuable to prevent damages to the oesophagus. By titration ablation power and/or duration for lesions deployed to the posterior wall of the LA, oesophageal wall oedema and lesion formation can be avoided [50]. Adjusting power settings and duration of ablation coupled with online lesion monitoring to further titrate duration of power delivery limits the risk of oesophageal damage.

## 8. Conclusion

With ICE, accurate 2D real-time and/or 3D imaging of the complex anatomy of LA and PVs is feasible. Intracardiac ultrasound improves the efficacy of electrophysiological interventional procedures by exactly identifying anatomical structures and integrating this information with electrophysiological parameters and/or 3D reconstructions of CT/MRI data. Early detection of periprocedural complications optimises emergency management. Implementation of ICE in ablation procedures of AF results in reduction of fluoroscopy/procedure time, potentially reduces complications [51], and improves outcome [10].
